# Adenosquamous carcinoma of the pancreas, a rare tumor entity: a case report

**DOI:** 10.1186/1757-1626-2-9129

**Published:** 2009-12-02

**Authors:** Pavlos Lampropoulos, Georgios Filippou, Evangelia Skafida, Thivi Vasilakaki, Nikolaos Paschalidis, Spiros Rizos

**Affiliations:** 1 Department of General Surgery, Piraeus General Hospital "Tzaneio", Afentouli & Tzani Str, Piraeus-Athens, 16345, Greece; 2 Department of Pathogical and Anatomical, Piraeus General Hospital "Tzaneio", Afentouli & Tzani Str, Piraeus-Athens, 16345, Greece

## Abstract

**Introduction:**

Adenosquamous carcinoma of the pancreas is a rare variant of exocrine pancreatic tumor. This type of tumor is extremely rare as only few similar cases have been described in the literature.

**Case presentation:**

We present a case of a 72 years old male patient who was admitted to the hospital complaining of epigastric pain and jaundice. Pancreatic carcinoma of the head was diagnosed and a pylorus preserving pancreaticoduodenectomy was performed.

**Conclusion:**

This type of cancer is a very aggressive tumor followed by a dismisal prognosis. Multimodality therapy seems to be a reasonable approach but more studies are needed, to propose the most effective treatment.

## Introduction

Pancreatic cancer is the 6th leading cause of death in Europe, while the incidence rate approaches the mortality rate [1]. Adenosquamous carcinoma is a rare entity of pancreatic cancer. Institutional reviews of autopsy and surgical specimens suggest an incidence of approximately 4% of all pancreatic neoplasms. It has been variously referred to as adenoacanthoma, mixed squamous and adenocarcinoma, and mucoepidermoid carcinoma. Only a few cases, 150 approximately, have been reported in the accessible literature, until 2008 [2]. Largest series published are from Rahemtullah et al and Kardon et al, with 14 and 25 cases respectively [3,4].

## Case presentation

**Figure 1:**
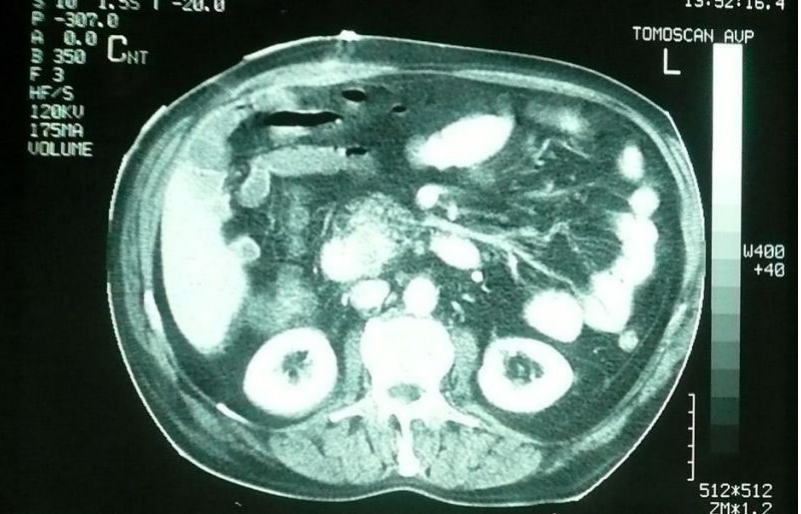
**CT scan of the patient showing the tumor**.

A 72-years-old Caucasian Greek male patient presented with epigastric pain of low intensity, jaundice and loss of appetite for the past 30 days. The patient was a heavy smoker (50 cig/day) and consumed alcohol (wine) occasionally, in small quantities. Physical examination revealed a distressed male patient with a body temperature of 37°C, blood pressure of 130/85 mmHg and pulse rate about 85/min. Painless jaundice was the presenting symptom and Courvoisier sign was present. The patient had no sings of ascites, or gastrointestinal obstruction. Hematocrit was 31%, white blood cells 10630/μL with neutrophil prevalence 77% and platelets 341.000/μL. Biochemical profile showed Tbil: 7.50 mg/dl, Dbil: 5.50 mg/dl, SGOT: 109 U/L, SGPT: 145 U/L, γGT: 517 U/L, ALP: 467 U/L, LDH: 503 U/L, CA125: 244, 50 U/L, CA19.9: 51.30 U/L. The patient was receiving digoxin and warfarin for chronic atrial fibrillation. Thin section computer tomography showed dilatation of the gallbladder and intra/extrahepatic biliary tree, while a 5 cm mass of the uncinate process of the pancreas was identified to invade the intrapancreatic portion of the bile duct, as well as the 2^nd ^and 3^rd ^portion of the duodenum (Figure 1). There was no extrapancreatic disease, no extension to the superior mesenteric artery and celiac axis. The superior mesenteric-portal confluence was patent. The decision for surgical intervention was taken, and a pylorus preserving pancreaticoduodenectomy was performed after exploratory laparoscopy. The patient had an uneventful postoperative course and discharged on the 11^th ^day.

**Figure 2:**
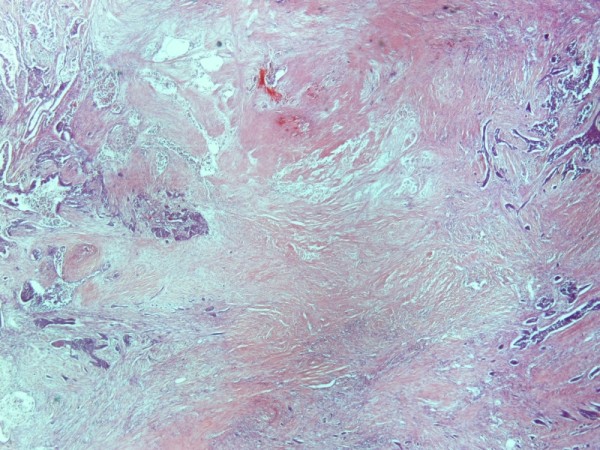
**(H&E ×20) Adenosquamous carcinoma of the pancreas with area of necrosis**.

**Figure 3:**
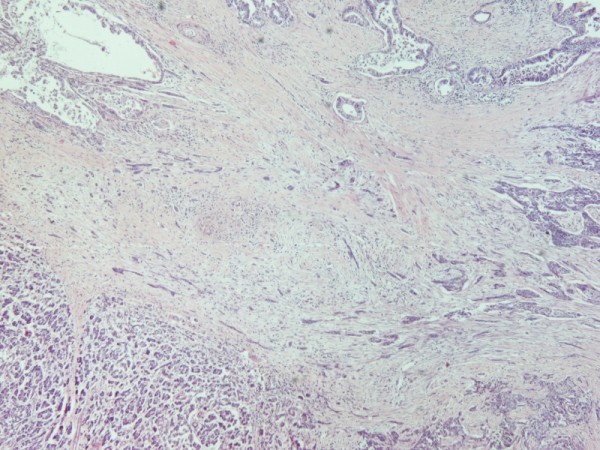
**(H&E ×40) Adenosquamous carcinoma of the pancreas**.

**Figure 4:**
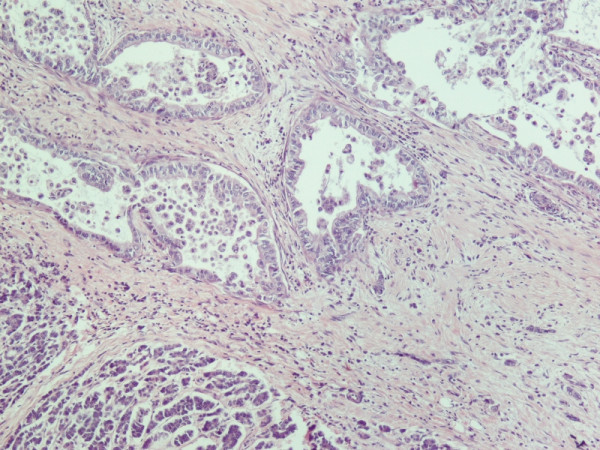
**(H&E ×100) Adenocarcinoma similar to ductal carcinoma**.

**Figure 5:**
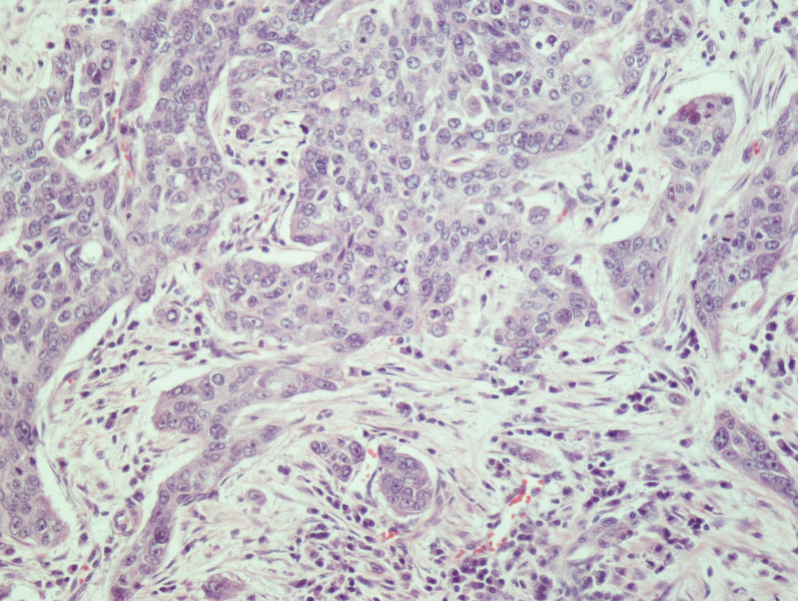
**(H&E ×200) Squamous cell carcinoma composed of irregular nests of polygonal cells with no gland formation**.

**Figure 6:**
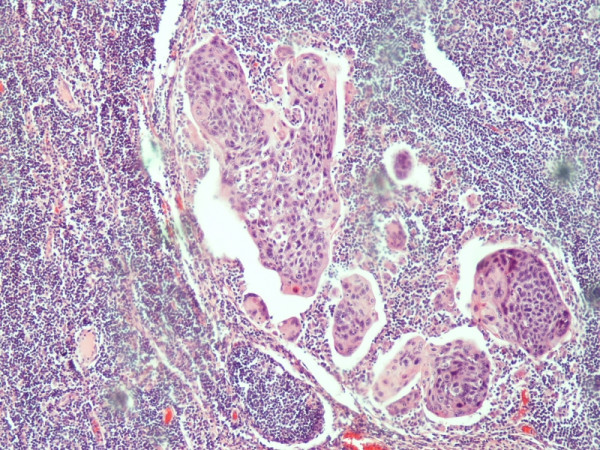
**(H&E ×100) Lymph node metastasis only from the squamous element**.

Histological evaluation of the pancreatic tumor showed an adenosquamous carcinoma which was extensively infiltrative with perineural invasion, involvement of peripancreatic lymph nodes and all the thickness of the duodenum wall. The tumor elicited an intense desmoplastic stromal reaction and areas of necrosis (Figure 2). Focal areas of high grade pancreatic intraepithelial neoplasia were seen. The tumor exhibited a biphasic malignant growth identified as, well to moderate differentiated adenocarcinoma and well to poorly differentiated squamous cell carcinoma (Figure 3). The adenocarcinoma component contained ductal or glandular structures with focal intracellular or extracellular mucin (Figure 4). Squamous differentiation was characterized by irregular and infiltrative nests or sheets of polygonal cells with distinct cellular borders, intercellular bridges, eosiniphilic cytoplasm and varying degrees of keratinization (Figure 5). These two different patterns could be seen separated topographically within the substance of the tumor or intimately admixed. Six of the 15 resected lymph nodes were positive for metastatic tumor which was composed of squamous carcinoma only (Figure 6).

**Figure 7:**
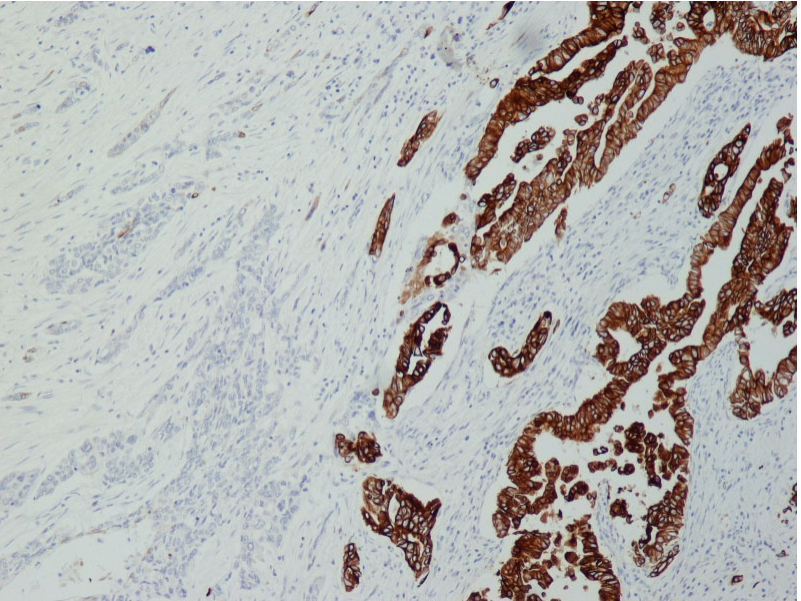
**(Ker7 ×100) Ker7 showed predilection for adenocarcinoma**.

**Figure 8:**
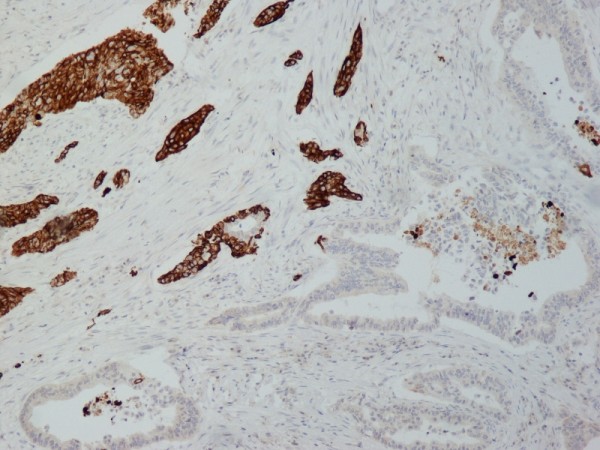
**(CK5/6 ×100) The squamous component was strongly immunoreactive with CK5/6, while the glandular component was negative**.

The immunohistochemical study showed that the tumor cells were positive for cytokeratin AE1 and AE2. Cam 5.2 and Ker 7 were reactive predominantly in the adenocarcinoma component and in few squamous cells (Figure 7). Immunoreactivity for CK 5/6 was restricted to the squamous component, while the glandular component was negative (Figure 8). A few number of tumor cells were immunoreactive with CEA and Ca 19-9. All tumor cells were negative for Ker 20, chromogranin and synaptophysin.

The patient received adjuvant therapy (5-FU in combination with radiation). He received 40 Gy in two 20 - Gy courses and 500 mg per m^2 ^5-FU by intravenous bolus delivered daily for the initial 3 days of each radiation course and then weekly. The patient 24 months postoperatively, remains disease free.

## Discussion

The incidence of adenosquamous carcinoma of the pancreas ranges among studies from 3% [5] to 11.1% [6], however the true incidence is not known since this is an aggressive tumor and most patients have not undergone surgery or autopsy. A few theories have been reported concerning the formation of adenosquamous carcinoma in an organ, where almost exclusively adenocarcinoma is the predominant cancer involved. One such theory postulates that a metaplastic squamous epithelium undergoes malignant transformation. Additionally, a preexisting adenocarcinoma could undergo malignant change and finally, a primitive cell may be capable of differentiating to either squamous or adeno component [6].

Preoperative diagnosis is often difficult, as there are no specific characteristics that can identify the squamous component in the lesion. However, Ga-67 citrate scintigraphy may be useful in detecting adenosquamous carcinoma of the pancreas [7]. On CT imaging, features suggestive of adenosquamous carcinomas of the pancreas include large infiltrative lesions and the presence of central necrosis [8]. Nabae et al. concluded that the presence of central necrosis in an infiltrative huge pancreatic tumor seems to be suggestive of the diagnosis of adenosquamous carcinoma of the pancreas [9]. The role of endoscopic US-guided FNA biopsy remains controversial.

Once the diagnosis is established either preoperative or postoperative, the question that rises refers to the best treatment modality for this aggressive tumor of the pancreas. From the literature reviewed, there is no study with significant statistical power to address that question. Smoot et al. demonstrated a longer survival for patients that can undergo an R0 resection. For R0 resection median survival was 14.4 months compared to 8 months for R1 and 4.8 months for patients undergoing palliative therapies [10]. Hsu JT et al. showed that the cumulative survival rates of 12 patients with pancreatic adenosquamous carcinomas, ranged from 1.12 to 22.42 months, with a median of 4.41 months. Additionally, patients with pancreatic adenosquamous carcinomas had shorter median survival compared to those with adenocarcinoma 6.51 months vs. 9.76 months [11]. Multidisciplinary treatments including aggressive surgery, intraoperative radiation therapy, and locoregional chemotherapy have also been reported to improve the survival of patients with adenosquamous carcinoma of the pancreas and to inhibit liver metastasis and local recurrence [12]. Shibagaki K et al. presented a case of successful use of chemoradiotherapy with low-dose cispastin and additional combined chemotherapy with S-1 and cisplatin for unresectable pancreatic adenosquamous carcinoma [13]. Tanaka N et al. suggested that combined chemotherapy with cytokines (IFN-a plus TNF-a) and 5-FU may be a therapeutic modality for advanced pancreatic cancer [14]. Wilkowski R, et al. concluded that gemcitabine and cisplatin can safely be combined with external beam radiation. This preoperative treatment approach is highly effective and appears to improve survival in patients whose tumors are rendered completely resectable [15]. However, there have been no sufficient studies that could propose a treatment modality for this rare tumor.

## Conclusion

It has become clear that this type of cancer has a dismissal prognosis. Even with aggressive therapies median survival is poor. More studies are needed to address the role of surgery and the use of adjuvant therapy, however this is quite difficult as this tumor entity is extremely rare. Since squamous cell carcinomas seem to respond satisfactory to chemoradiotherapy regimens based on 5-FU, this combination could be potentially used as adjuvant therapy in patients with resectable squamous cell pancreatic cancer, or as palliative therapy for unresectable tumors. R0 resection remains the gold standard for those individuals who are fit enough to undergo such a major operation.

## Consent

Written informed consent was obtained from the patient for publication of this case report and any accompanying images. A copy of the written consent is available for review by the Editor-in-Chief of this journal.

## Competing interests

The authors declare that they have no competing interests.

## Authors' contributions

PL participated in the patient's treatment, made the literature search, contributed in the writing and the revision of the draft. GF contributed in the literature search, co wrote the draft and made the revision. ES carried out the pathological examination and contributed in the writing of the draft. TV participated in the diagnosis of the case and in the presentation of its pathology, contributed in the pathological examination and in the revision of the draft. NP participated in the patient's treatment, had the concept and the coordination of the case report and contributed in the revision. SR contributed in the revision and had the final approval of the draft. All authors read and approved the final manuscript.
